# Physician Gender Preference Amongst Females Attending Obstetrics/Gynecology Clinics

**DOI:** 10.7759/cureus.15028

**Published:** 2021-05-14

**Authors:** Bismah Riaz, Nawabzada Zeerak Farhat Sherwani, Syed Hashim Ali Inam, Muhammad Yasir Rafiq, Saman Tanveer, Anum Arif, Mohammad Abdullah, Hamza Jamil

**Affiliations:** 1 Internal Medicine, Combined Military Hospital (CMH) Lahore Medical and Dental College, Lahore, PAK; 2 Epidemiology and Public Health, Army Medical College, Rawalpindi, PAK; 3 Internal Medicine, Dudley Group of Hospitals, Dudley, GBR; 4 Internal Medicine, Army Medical College, Lahore, PAK; 5 Vascular Surgery, Combined Military Hospital (CMH) Lahore Medical College, National University of Medical Sciences (NUMS), Lahore, PAK; 6 Stroke, Russells Hall Hospital, Dudley, GBR; 7 Internal Medicine, Army Medical College, Rawalpindi, PAK

**Keywords:** gender bias, male gynecologists, female preference, discrmination, patient comfort

## Abstract

Introduction: The objective of our study was to explore the views and perceptions of female patients attending the obstetrics and gynecology (OB/GYN) outpatient department towards the gender of their healthcare provider, to look for any preference that might exist in this regard, and to highlight any discrimination towards male obstetricians/gynecologists.

Material and methods: A cross-sectional study was conducted from November 2020 to March 2021 at Combined Military Hospital, Lahore. A total of 280 female patients were included in the study and interviewed consecutively. A self-designed questionnaire was administered. Data were analyzed using Statistical Package for Social Sciences (SPSS) version 22 (IBM Corp., Armonk, NY). Chi-square test was used to determine for any statistical significance and p≤0.05 was considered significant. Numerical data were represented as percentages.

Results: Over 280 female patients participated in our study, out of which 132 were married and 148 were single. Thirteen of these patients were uneducated, 40 had completed high school and 227 patients had obtained a bachelor's level of education; 120 patients were aged 15-25 years, 95 patients were aged 26-35 years, 30 patients were aged 36-45 years, and 35 patients were aged >46. Women with less education preferred to be seen by a female obstetrician/gynecologist, whereas those with higher education were less biased (p=0.0001). Married patients preferred to be seen by female obstetrician/gynecologists as compared to single patients (p=0.0004). A significant proportion of females were impartial in terms of physician competence but those who did have a preference preferred female obstetrician/gynecologist based on three significant variables: competence, rapport building and empathy, and personal comfort.

Conclusion: A female gender preference exists in obstetrics and gynecology clinic attendees. This is significant in those who are married and those with no formal education. Overall, a significant proportion of women feel a higher comfort level with female doctors and find it easier to discuss their medical issues and develop rapport.

## Introduction

In the past, gynecology was a male-dominated field, but recently, amongst prospective male doctors, only one in 500 ends up choosing a career in obstetrics/gynecology (OB/GYN) [1,2]. The reason behind this is understudied. Over the last decade, patients’ preference for their gynecologist's gender has changed. While some patients attribute equal competence in emotional, professional, and interpersonal aspects to both genders, there are some who demonstrate a preference towards female obstetricians/gynecologists [3]. In low socioeconomic countries such as ours, cultural reasons, level of patients’ education, and religious factors are the main contributors to discrimination against male gynecologists [4]. Due to this, male doctors can feel unaccepted in OB/GYN settings which limits not only their exposure and learning but also discourages them from considering OB/GYN as a specialty.

Preference affects physician-patient relationships which can manipulate various clinical outcomes including patient satisfaction, compliance to treatment and follow-up appointments, and in some cases, the general health of the patient. Some factors that contribute to patients preferring one gender over the other include ease of communication from the patient’s end when expressing their medical issues, empathy, rapport building, and religious barriers when it comes to gynecological examination and procedures.

The concept of doctor gender preference has been studied widely in primary practice [5-8]. However, it has not been explored much in the field of OB/GYN. In our study, we have aimed to explore the views and perceptions of female patients attending the OB/GYN outdoor patient department towards the gender of their healthcare provider and to highlight any discrimination towards male obstetricians/gynecologists that may exist.

## Materials and methods

A cross-sectional study was conducted from November 2020 to March 2021 at Combined Military Hospital, Lahore. This center is a tertiary referral center for a large population. The study participants were all females, aged 18 years and above, attending OB/GYN outdoor patient department who consented to participate in the study. A total of 280 female attendees met the inclusion criteria and were interviewed consecutively. Patients who did not consent, or with whom a communication barrier existed during the interview were excluded from the study.

A self-designed questionnaire was used which consisted of two parts: the first part included consent, patient’s demographics, and level of education, and the second part included questions about the preferred gender of obstetrician-gynecologist and the reasons behind their preference. After a thorough literature review, three main reasons for doctor gender preference were added to the questionnaire - competence, rapport building and empathy, and personal comfort. To avoid any misinterpretation of the questionnaire, the interview was conducted by one of the authors, making sure any ambiguity was cleared out then and there.

Prior to initiation of the study, approval was sought from the hospital’s ethical review board and informed consent was sought from all patients when inviting them to participate in the study. The study was conducted in accordance with the principles laid down in the declaration of Helsinki. Data were analyzed using Statistical Package for Social Sciences (SPSS) version 22 (IBM Corp., Armonk, NY). Chi-square test was used to determine for any statistical significance and p≤0.05 was considered significant. Numerical data were represented as percentages.

## Results

A total of 280 females consented to participate in the study. Characteristics of the study population have been described in Table 1.

**Table 1:** Characteristics of the study population

		Prefer male obstetrician/gynecologist	Prefer female obstetrician/gynecologist	Neutral	Total (n)	P-value
Marital status	Married	5	109	18	132	<0.01
	Single	10	96	42	148	
Level of education	Uneducated	1	10	2	13	<0.01
	High school	0	40	0	40	
	Bachelors	14	155	58	227	
Age (in years)	15–25	7	82	31	120	<0.01
	26–35	7	70	18	95	
	36–45	0	22	8	30	
	>46	1	31	3	35	

We noted that women with less education preferred to be seen by a female obstetrician/gynecologist (p=0.001), and those with higher education were less biased. Similarly, we also found a significant association between gender preference and marital status (p=0.004).

We attempted to explore participant’s perceptions of various characteristics about their physician. We asked about three key characteristics - competence, empathy and rapport, and personal comfort. The findings are elaborated on in Table 2.

**Table 2:** Perception about male and female gynecologists as answered by the study population

		Frequency	Percentage	P-value
Who do you think is a more competent obstetrician/gynecologist?	Male	21	7.5%	<0.01
	Female	81	28.9%	
	Neutral	178	63.6%	
Who do you think is better at rapport building and empathic?	Male	34	12.1%	<0.01
	Female	151	53.9%	
	Neutral	95	33.9%	
Whom would you feel comfortable to consult for your issue(s)?	Male	7	2.5%	<0.01
	Female	239	85.4%	
	Neutral	34	12.1%	

In a summary of the above findings, we observed that a significant proportion of females were impartial in terms of physician competence, but those who did have a preference, preferred a female obstetrician/gynecologist. Similarly, in terms of rapport building and empathy, and feeling comfortable expressing their medical problems, most of the patients preferred a female doctor.

All of the three variables were significant in determining the choice of the gender of the doctor (p<0.01) because all three had a significant impact on the choice.

## Discussion

In the last century, OB/GYN was dominated by males [1]. However, in recent years there has been a shift towards females. The choice for a gynecologist, or any physician for that matter, should ideally be determined by credentials and career performance rather than by the doctor’s gender. Therefore, research on this issue is encouraged to find the reasons for the bias and to eradicate any misconceptions. Any specialty should cater equally for either gender, but the efforts of the system fail when the problem rests with patients’ preference.

Some studies show that preference for a female gynecologist was mostly seen in younger women [9,10]. However, in our study, age had no significant relation with preference (p=0.036). Similarly, Chandler et al. [11] did not notice any variation in gender. We found that the level of education had a significant relation with the choice of gender (p=0.002) such that, women with no formal education were more inclined towards being seen by a female gender doctor. Feng et al. [12] did a similar regional study, but education was statistically insignificant in their study.

Personal comfort of patients was the biggest reason females want to be seen by the same-gender doctor. The matter of personal comfort comes to play in intimate settings where either an internal examination is warranted or something of a personal or sensitive nature is to be disclosed. Such aspects are seen on a regular basis in OB/GYN. This is supported by a systemic review by Janssen and Lagro-Janssen [13]. When asked about competence, most of the patients were neutral (63.6%). This is an aspect that has not been explored much. Although several excerpts from the literature state that patients would rate competence over gender, reports on which gender does the patient considers more competent are scarce. Lastly, rapport building and empathy was also perceived to be a trait more commonly affiliated with the female gender. This is in accordance with Chandler et al. who found that 74% of patients in their population stated that female gynecologists “understand the problem better” [11]. In comparison, we noted that Howell et al. studied 73 obstetric patients during their postpartum hospital stay and found that only one-third of their patients preferred female obstetricians/gynecologists [14]. Similarly, Schnatz et al. (n=72) also found that 80.3% of their participants had no gender preference [15]. Lastly, Johnson et al. (n=264) also noted in their survey that the majority of females did not select their obstetrician/gynecologist solely based on gender [16].

There were certain limitations to our study. The study was a single-center experience and may not represent patient preferences elsewhere. The study participants were females and their choices and preferences are not representative of choices by male patients, who also visit Gynecology clinics for their complaints. Moreover, some of the reasons for stated preference such as empathy cannot be validated on any objective scale. We used a closed-ended questionnaire; the use of an open-ended questionnaire may allow patients to express the full scope of their feelings and reactions to the questions asked. Religious and cultural obligations were not addressed in detail in this study.

## Conclusions

A female gender preference exists in OB/GYN clinic attendees. This is significant in those who are married and those with no formal education. Overall, a significant proportion of women feel a higher comfort level with female doctors and find it easier to discuss their medical issues and develop rapport. It is critical that we examine this discrimination and analyze its effects on anyone’s preference in learning about OB/GYN and considering it as a career. A female-dominated specialty encourages the public perception that women alone are only qualified enough to care for women.
